# Topical Application of Butyl Flufenamate Ointment Promotes Cranial Defect Healing in Mice by Inducing BMP2 Secretion in Skin Mesenchymal Stem Cells

**DOI:** 10.3390/cells11223620

**Published:** 2022-11-15

**Authors:** Fan Yang, Xuenan Liu, Donghao Wei, Yuan Zhu, Feilong Wang, Xuejiao Liu, Fanyu Yan, Xiao Zhang, Yunsong Liu

**Affiliations:** 1 Department of Prosthodontics, Peking University School and Hospital of Stomatology, National Laboratory for Digital and Material Technology of Stomatology, Beijing Key Laboratory of Digital Stomatology, National Clinical Research Center for Oral Diseases, 22 Zhongguancun South Avenue, Beijing 100081, China; 2 Department of Oral Implantology, Peking University School and Hospital of Stomatology, National Laboratory for Digital and Material Technology of Stomatology, Beijing Key Laboratory of Digital Stomatology, National Clinical Research Center for Oral Diseases, 22 Zhongguancun South Avenue, Beijing 100081, China; 3 Shanghai Key Laboratory of Craniomaxillofacial Development and Diseases, Shanghai Stomatological Hospital, Fudan University, Shanghai 200437, China

**Keywords:** butyl flufenamate ointment, mouse cranial defect, mouse-skin mesenchymal stem cell, BMP2

## Abstract

Bone defects and fractures heal slowly compared with injuries to other tissues, creating a heavy burden for patients, their families, and society. Alongside conventional treatment methods for fractures and bone defects, adjuvant therapies play an important but underappreciated role. In a previous study, we found that systemic administration of flufenamic acid promoted osteogenesis in vivo, but its side effects limited the application of our findings. In the present study, we assess the effects of external butyl flufenamate ointment on the healing of cranial defects in mice. We found that application of butyl flufenamate ointment on the surface of the skin accelerated the healing of cranial defects in mice by promoting BMP2 secretion from mouse-skin mesenchymal stem-cells. These findings indicate that butyl flufenamate ointment has potential therapeutic value for treating superficial fractures or bone defects while avoiding the toxicity and side effects of systemic medication, representing a safe and convenient adjuvant therapy to promote healing of superficial bone defects and fractures.

## 1. Introduction

Bone defects caused by fractures, tumors, congenital malformation, trauma, and infection are common in the clinic and can cause serious harm to patients’ physical and mental health [1]. Since the healing speed of bone is far slower than that of other tissues, bone defects and fractures also impose a heavy economic burden on patients’ families and society [2,3]. Alongside conventional treatment methods for fractures and bone defects, adjuvant therapies play an important but underappreciated role [3].

Local administration has several benefits compared with systemic administration, including decreased side effects and toxicity, greater convenience, and improved patient compliance [4,5]. Thus, transdermal drug delivery has become an attractive field [6,7]. Fractures and bone defects in many body parts are relatively superficial, such as the hands, feet, maxillofacial region, knee joint, and elbow joint. The use of external drugs for fractures and bone defects at these sites may have an auxiliary effect in accelerating healing.

As a member of the non-steroidal anti-inflammatory drug family, flufenamic acid (FFA) is a representative of fenamic acid. It is commonly used in the clinic to treat inflammatory diseases, such as rheumatic arthritis [8,9]. Oral administration of FFA is no longer recommended because it has a series of non-negligible side effects, such as kidney and digestive tract damage [10,11,12,13]. Thus, FFA is currently only applied externally. Butyl flufenamate ointment is a commonly used commercial form of FFA. In a previous study, we found that a low concentration of FFA suppressed bone loss in ovariectomized and aged mice by systemic administration [14,15]. However, when taking the side effects of FFA into consideration, the clinical value of our previous finding is limited. Whether FFA can be used as an adjuvant therapy for bone defects and fractures is unknown. In recent years, several studies have examined local administration of FFA [11,16,17], but there have not been any reports on transdermal administration of FFA in the treatment of bone defects.

In the present study, we aimed to explore whether the topical application of butyl flufenamate ointment on the skin can promote healing of cranial defects in mice. Our results showed that topical application of butyl flufenamate ointment accelerated the healing of cranial defects in mice. Mechanistically, butyl flufenamate ointment accelerated healing by promoting BMP2 secretion from mouse-skin mesenchymal stem-cells (mSMSCs). This study provides valuable data on the potential application of butyl flufenamate ointment to treat superficial bone defects, representing a safe and convenient adjuvant therapy to promote healing.

## 2. Materials and Methods

### 2.1. Animals

All animals used in this study were obtained from Vital River Corporation (Beijing, China). The mice were kept in a pathogen-free facility on a 12:12-h light: dark cycle with water and food provided ad libitum. The Institutional Animal Care and Use Committee of the Peking University Health Science Center (LA2019019) approved the in vivo experiments in the present study, and all experiments were performed under relevant guidelines.

### 2.2. Isolation and Culture of mSMSCs

Collage I, dispase V, fetal bovine serum (FBS), modified Eagle’s medium (MEM), and 100× penicillin/streptomycin solution were purchased from Gibco (Grand Island, NY, USA). Other reagents and materials were obtained from Sigma-Aldrich (St. Louis, MO, USA) unless stated otherwise.

Eight-week-old female C57BL6 mice were used to isolate mSMSCs. After the mice were sacrificed, the skin on their backs was prepared and disinfected. An area of skin on the back approximately 1.5 × 1.5 cm^2^ in area was carefully removed. The skin was then disinfected with 75% ethanol and washed with PBS. Then, the skin was cut as finely as possible and digested using a mixture of 2 mL collagenase I (4 mg/mL) and 2 mL dispase V (8 mg/mL) at 37 °C for approximately 1.5 h. After adding 4 mL MEM, the mixture was centrifuged at 1500 rpm for 5 min. The sediment was then resuspended in medium, filtered through a 70 μm sieve, and seeded in 100 mm dishes. The cells were then cultured in a 5% CO_2_ atmosphere at 37 °C in proliferation medium (PM) consisting of MEM, penicillin/streptomycin, and 20% (v/v) FBS. After 24 h, the cells were washed twice with PBS, and new PM was added. Cells from passages 2–4 were used in subsequent experiments.

### 2.3. Osteogenic, Adipogenic, and Chondrogenic Induction of mSMSCs

The osteogenic medium (OM) was prepared by mixing MEM, 10% (*v*/*v*) FBS, penicillin/streptomycin, 10 mM β-glycerophosphate, 10 nM dexamethasone, and 0.2 mM L-ascorbic acid. The adipogenic medium (AM) was prepared by mixing MEM, 10% (*v*/*v*) FBS, penicillin/streptomycin, 100 nM dexamethasone, 200 μM indomecin, 10 μM insulin, and 500 μM 3-isobutyl-1-methylxanthine. The osteogenic medium (OM) was prepared by mixing MEM, 10% (*v*/*v*) FBS, penicillin/streptomycin, 10 nM dexamethasone, 10 mM β-glycerophosphate, and 0.2 mM L-ascorbic acid. The chondrogenic medium (CM) was prepared by mixing MEM, 10% (*v*/*v*) FBS, penicillin/streptomycin, 1 μg/mL ascorbic acid, 100 nM dexamethasone, 1% sodium pyruvate, 10% insulin–transferrin–selenium (ITS-Premix), and 10 ng/mL mouse transforming growth factor β1 [18].

### 2.4. Co-Culture of Mouse Bone Marrow Mesenchymal Stem Cells (mBMMSCs) with mSMSCs Using Transwell Membranes

Primary mBMMSCs were obtained from Pricella (Wuhan, China), which were derived from C57/BL6 mice. Cells from passages 3–5 were used in the experiments. The mBMMSCs were first seeded in the bottom of wells of a 6-well plate. A transwell permeable support (pore size 0.4 μm) was placed in each well. The mSMSCs were then seeded in the upper part of each well without physical contact with the mBMMSCS.

### 2.5. Preparation of a Concentrated FFA Solution

For the FFA treatment of cells in the in vitro experiments, we first dissolved FFA in DMSO to obtain a concentrated 50 mM (1000×) FFA solution, which was shown to have no significant effect on cells [14].

### 2.6. ELISA

The concentration of BMP2 secreted into the culture medium from mSMSCs was measured using an ELISA kit (Cloud-Clone, Katy, TX, USA) according to the manufacturer’s instructions.

### 2.7. Alkaline Phosphatase (ALP) Staining and Quantification

After 7 days of culture in PM or OM, the cells were washed with PBS, fixed in ethanol, and washed again with PBS. An ALP staining kit (CWBIO, Beijing, China) was used to assess ALP expression, and the images of stained cells were then scanned. ALP activity was quantified as described previously [14]. Briefly, the cells were lysed using 1% Triton X-100 on ice. A BCA protein assay kit (Pierce Thermo Scientific, Waltham, MA, USA) was used to measure the total protein concentration. ALP activity was measured using an ALP assay kit (Nanjing Jiancheng Bioengineering Institute, Nanjing, China), and the ALP activity level was normalized to the total protein concentration in each sample.

### 2.8. Alizarin Red S (ARS) Staining and Quantification

Cells were cultured with PM or OM, respectively, and after 14 days of culture, cells were washed twice with PBS, fixed with ethanol, and washed three times with distilled water. First, 2% Alizarin Red S (ARS) staining solution was prepared, added to the cell culture well plate, waited for 10–20 min, and the staining image was scanned. Add 100 mM cetylpyridine chloride solution to the well plate to dissolve the stained cells for ARS quantification. Determine the optical density of each hole at 562 nm by spectrophotometry.

### 2.9. Oil Red O Staining

Oil red O staining was performed according to the method tested very frequently by our team [15]. Briefly, cells were cultured using PM and AM, respectively, and after 21 days of culture, cells were fixed with 10% formalin, after which the formalin was washed with 60% isopropanol. Briefly, 21 days after culture in PM or AM, the cells were fixed in 10% formalin, rinsed with 60% isopropanol, and 0.3% oil Red O staining solution was added to the cell well plates for staining. Next, the cells were washed in distilled water, viewed under a microscope, and photographed.

### 2.10. Alcian Blue Staining

At 21 days after chondrogenic induction, the cells were washed with PBS and fixed in 10% formalin. After 1 h, the cells were washed with distilled water and stained with Alcian blue solution overnight in the dark. The cells were washed with de-staining solution and PBS, followed by scanning [18].

### 2.11. Quantitative Real-Time Reverse-Transcription PCR (qRT-PCR)

Trizol reagent (Invitrogen, Carlsbad, CA, USA) was used to extract total RNA from mBMMSCs or mSMSCs. Next, the PrimeScript RT Reagent Kit (Takara, Tokyo, Japan) was used to reverse transcribe the RNA into cDNA. Finally, SYBR Green Master Mix (Roche Applied Science, Mannheim, Germany) was used for quantitative real-time PCR, which was performed on the ABI Prism 7500 real-time PCR system (Applied Biosystems, Foster City, CA, USA). Glyceraldehyde-3-phosphate dehydrogenase (*gapdh*) was chosen as the internal reference gene. The primer sequences used were as follows: *gapdh* (forward) 5′-TCACTCAAGATTGTCAGCAA-3′ and *gapdh* (reverse) 5′-AGATCCACGACGGACACATT-3′; *runx2* (forward) 5′-TAAGAAGAGCCAGGCAGGTG-3′ and *runx2* (reverse) 5′-TGGCAGGTACGTGT GGTAGT-3′; *bglap* (forward) 5′-TGCTTGTGACGAGGTATCAG-3′ and *bglap* (reverse) 5′-GTGACATCCATACTTGCAGG-3′; *osx* (forward) 5′-TCACTTGCCTGCTCTGTTCC-3′ and *osx* (reverse) 5′-GCGGCTGATTGGCTTCTTCT-3′; *bmp2* (forward) 5′-ACTTTTCTCGTTTGTGGAGC-3′ and *bmp2* (reverse) 5′-GAACCCAG GTGTCTCCAAGA-3′; *bmp4* (forward) 5′-GAGGAGGAGGAAGAGCAGAG-3′ and *bmp4* (reverse) 5′-TGGGATGTTCTCCAGATGTT-3′.

### 2.12. Cranial Defect Surgery in Mice

Twenty 8-week-old female C57BL6 mice were used for the in vivo experiments. To minimize suffering, mice were anesthetized by intraperitoneal injection of 1% sodium pentobarbital (50 mg/kg), followed by craniotomy and periosteum separation. Next, a critical-sized defect (diameter: 4 mm) was made at the calvarium avoiding the bone seam by a trephine bur (Hager Meisinger GmbH, Neuss, Germany) under low-speed drilling. During the operation, a large amount of saline should be continuously used for flushing to prevent the adverse effect of high temperature on the surrounding bone. During the operation, care should be taken to avoid damaging the dura mater and brain of mice. Finally, the incision was closed with 5-0 suture.

### 2.13. Application of Butyl Flufenamate Ointment to the Skin Surface of the Cranial Defect Area

After surgery, mice with round cranial defects (diameter: 4 mm) were randomly divided into the Vaseline and FFA groups. Mice in the Vaseline group were given Vaseline, as a control, while mice in the FFA group were given butyl flufenamate ointment. Either Vaseline or butyl flufenamate ointment was administered to the skin over the defect area daily, beginning on the fifth day after cranial-defect surgery, when the wound on the skin surface had healed.

### 2.14. Micro-Computed Tomography (CT) and Bone Morphometric Analysis

Mice were sacrificed at 6 and 8 weeks after surgery by careful use of excessive anesthesia, and the skulls of the mice were harvested. Cranial bones were fixed with 10% formalin, and the surface of the sample was washed with 10% sucrose solution for formalin. Then, the cranial bones were scanned using a micro-CT with a resolution of 8.82 μm, tube voltage of 60 kV, tube current of 500 μA, and exposure time of 1500 ms. Three-dimensional (3D) reconstruction images were obtained using multimodal 3D visualization software (Inveon Research Workplace; Siemens, Munich, Germany) based on two-dimensional images. Bone volume/total volume (BV/TV) and bone mineral density (BMD) parameters in the defect area were calculated by Inveon Research Workplace (Siemens) [19].

### 2.15. Statistical Analysis

The results are expressed as means ± standard deviation. SPSS Statistics 20.0 software (SPSS Inc., Chicago, IL, USA) was used for the statistical analyses. Analysis between sample groups was conducted using independent two-tailed Student’s *t*-tests, one-way ANOVA, and Tukey’s post hoc test. A significance level of 5% was used for all tests.

## 3. Results

### 3.1. Application of Butyl Flufenamate Ointment to the Skin Surface Promoted Cranial Defect Healing in Mice

**Figure 1:**
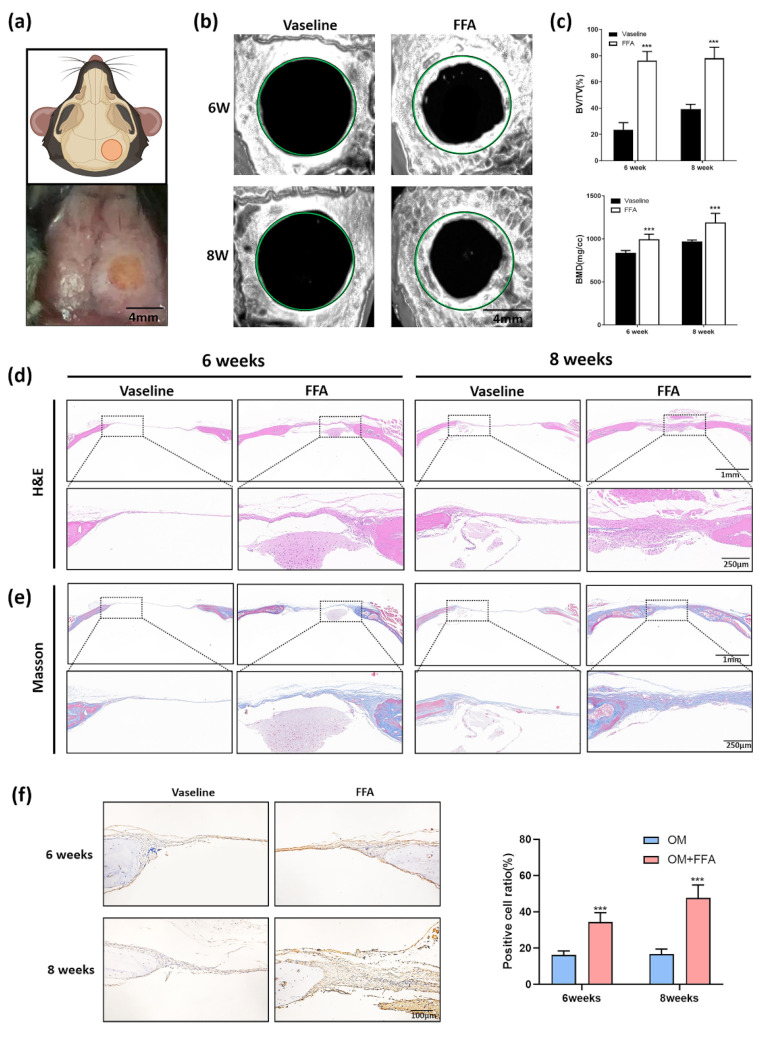
Application of butyl flufenamate ointment to the skin surface promoted cranial-defect healing in mice. (**a**) Schematic diagram and clinical operation picture of mice cranial defects. Scale bar = 4 mm. (**b**) Micro CT 3D reconstruction images of cranial defects showing that butyl flufenamate ointment promoted new bone formation after 6 weeks. Treatment with butyl flufenamate ointment had a stronger effect after 8 weeks than after 6 weeks. Scale bar = 2 mm. (**c**) Improvements in BV/TV and BMD in the defect areas after butyl flufenamate compared with Vaseline treatment, especially after 8 weeks. (**d**,**e**) Hematoxylin–eosin and Masson’s trichrome staining images of the cranial-defect area in each group. Scale bar = 1 mm and 250 μm. (**f**) IHC staining (OCN) of the cranial-defect area in different groups. Scale bar = 100 μm. *** *p* < 0.001.

In a previous study, we found that FFA suppressed osteoporosis caused by ovariotomy and aging in mice, indicating that FFA has potential therapeutic value for bone metabolism diseases. As a member of the non-steroidal anti-inflammatory drug family, FFA has been approved by the FDA. It is typically applied topically, commonly in the form of butyl flufenamate ointment. We applied butyl flufenamate ointment to the skin over cranial defects in mice to explore whether it promoted healing of the defect. Figure 1a shows the schematic diagram and clinical operation picture of mice cranial defects. Twenty 8-week-old female C57 mice with round cranial defects (4 mm diameter) were randomly divided into two groups: Vaseline and FFA. Cranial bone samples were harvested 6 or 8 weeks after surgery. According to the micro-CT 3D reconstruction images, there was little new bone formation in the defect area in the Vaseline group after 6 or 8 weeks (Figure 1b). However, butyl flufenamate ointment significantly promoted the formation of new bone in the defect area, with a stronger effect after 8 weeks compared with 6 weeks (Figure 1b). Compared with Vaseline, butyl flufenamate ointment improved BV/TV and BMD in the defect area, especially after 8 weeks of treatment (Figure 1c). Hematoxylin–eosin (Figure 1d) and Masson’s trichrome (Figure 1e) staining revealed a greater number of new osteoids in the FFA group after 6 and 8 weeks, which was consistent with the micro-CT results. Immunohistochemical (IHC) staining of osteocalcin (OCN) (Figure 1f) showed a greater area of positivity in the FFA than Vaseline treatment group, which confirmed previous findings.

### 3.2. Isolation and Stemness Determination of mSMSCs

Butyl flufenamate ointment is easily absorbed by the skin, where 95% of it accumulates. This means that butyl flufenamate cannot directly affect the cranial bone. Thus, we inferred that butyl flufenamate ointment promotes cranial-defect healing via effects on the skin.

**Figure 2:**
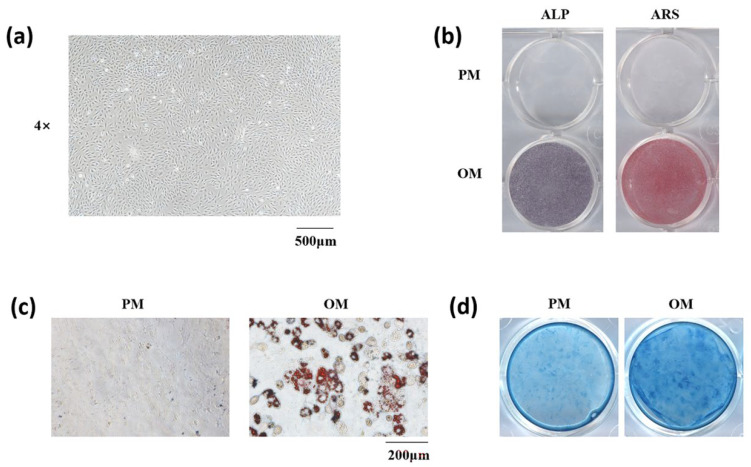
Isolation and stemness determination of mSMSCs. (**a**) Images of mouse mesenchymal stem cells. Scale bar = 500 μm. (**b**) ALP staining and ARS staining after osteogenic induction of mSMSCs. (**c**) Oil red O staining after adipogenic induction of mSMSCs. Scale bar = 200 μm. (**d**) Alcian blue staining after chondrogenic induction.

We first isolated mSMSCs from female C57 mice (Figure 2a). Next, to identify the stemness of the obtained cells, we treated them with OM, AM, and CM to confirm their osteogenic, adipogenic, and chondrogenic differentiation capacities, respectively. ALP and ARS staining showed that the cells could differentiate into osteoblasts (Figure 2b). Meanwhile, oil red O (Figure 2c) and Alcian blue (Figure 2d) staining showed that the cells can differentiate into adipocytes and chondrocytes.

### 3.3. Co-Culture with mSMSCs Promoted Osteogenesis in mBMMSCs in the Presence of a Low Concentration of Flufenamic Acid

**Figure 3:**
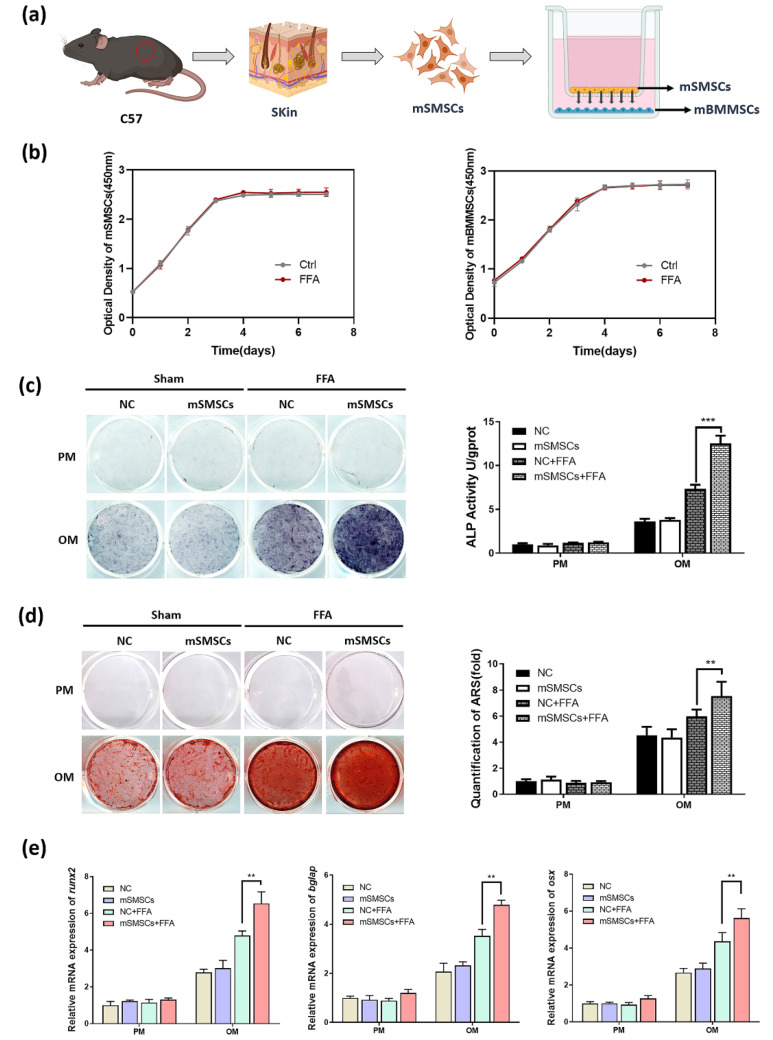
Co-culture with mSMSCs promoted osteogenesis of mBMMSCs in the presence of a low concentration of FFA. (**a**) The process of obtaining mSMSCs and the pattern of mSMSC and mBMMSC growth in co-culture. (**b**) Cell proliferation values for two groups, obtained using the Cell Counting Kit-8 on days 1, 2, 3, 4, 5, 6, and 7. (**c**) ALP staining and quantification showing that co-culture with mSMSCs markedly increased ALP activity in mBMMSCs in the presence of FFA. (**d**) ARS staining and quantification showing that co-culture with mSMSCs significantly accelerated mBMMSC mineralization in the presence of FFA. (**e**) qRT-PCR showing that co-culture with mSMSCs increased the mRNA expression of *runx2*, *bglap*, and *osx* in mBMMSCs in the presence of FFA. ** *p* < 0.01, *** *p* < 0.001.

To verify whether FFA promotes osteogenesis in mBMMSCs by affecting the skin, we co-cultured mBMMSCs with mSMSCs. To mimic the pattern of the two cell types in vivo, we used a cell contact-independent, soluble mediator-driven co-culture system wherein mSMSCs (upper) and mBMMSCs (lower) were physically separated by a semipermeable transwell membrane (Figure 3a) [20]. The cell viability of mSMSCs and mBMMSCs was detected by the CCK-8 method (Figure 3b). There was no significant difference in OD450 values between the two groups, which showed that FFA at a concentration of 50 mM did not affect the proliferation and growth of mBMMSCs and SMSCs. After 7 days of osteogenic induction, ALP staining and quantification showed that FFA at a low concentration increased ALP activity, which was consistent with our previous study using human stem cells (Figure 3c). Co-culture with mSMSCs had no effect in the absence of FFA in the culture medium but significantly enhanced ALP activity in the presence of FFA (Figure 3c). ARS staining and quantification on day 14 showed similar results to those of ALP staining and quantification (Figure 3d). We used qRT-PCR to assess the mRNA expression of several markers (*runx2*, *bglap*, and *osx*) during osteogenesis (Figure 3e). The results were consistent with the results of ALP and ARS staining and quantification, indicating that co-culture with mSMSCs promotes osteogenesis of mBMMSCs in the presence of low concentrations of FFA.

### 3.4. Butyl Flufenamate Ointment Accelerated Cranial Defect Healing in Mice by Promoting BMP2 Secretion from mSMSCs

**Figure 4:**
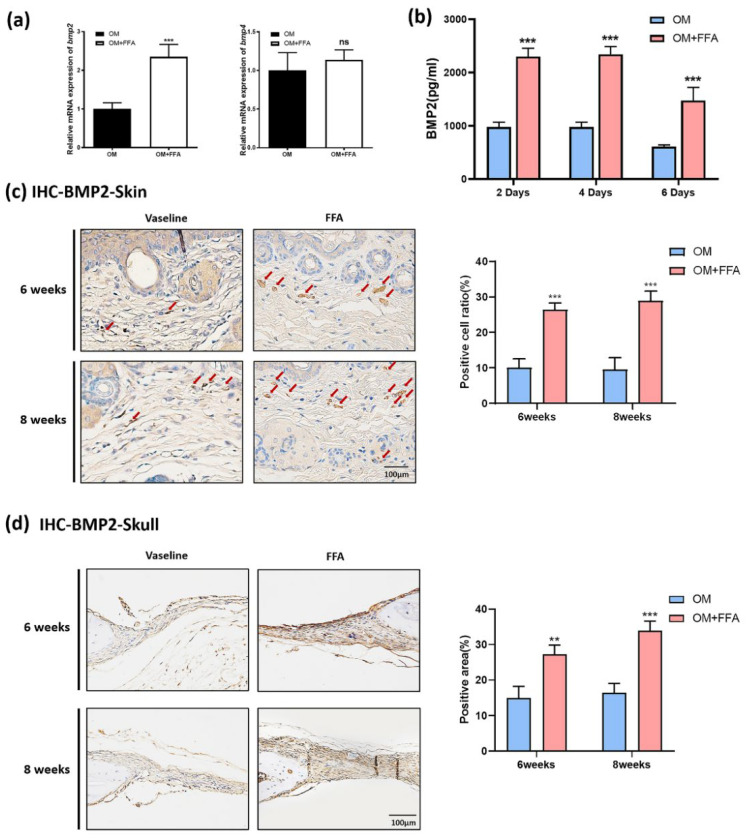
Butyl flufenamate ointment accelerated cranial-defect healing in mice by promoting BMP2 secretion in mSMSCs. (**a**) qRT-PCR showing that FFA increased the mRNA expression of *bmp2* but not *bmp4* in mSMSCs. (**b**) ELISA showing that FFA increased BMP2 secretion in mSMSCs at days 2, 4, and 6. (**c**) IHC staining of BMP2 in the skin in the cranial-defect area in different groups and the percentage of positively stained cells calculated according to the IHC images. Scale bar = 100 μm. (**d**) IHC staining of BMP2 in the cranial-defect area in different groups and the percentage of the positive staining area calculated according to the IHC images. Scale bar = 200 μm. ** *p* < 0.01, *** *p* < 0.001, ns no significance.

Next, to further explore how mSMSCs mediated the effects of butyl flufenamate on cranial-defect healing, we evaluated the expression of several key secretory factors related to osteogenesis in mSMSCs by qRT-PCR. The results showed that FFA treatment significantly increased the expression of *bmp2* but not *bmp4* (Figure 4a). We performed ELISA to determine the BMP2 concentration in the culture medium of mSMSCs. ELISA demonstrated significantly higher concentrations of BMP2 present in the culture medium in the presence of FFA on days 2, 4, and 6. Moreover, BMP2 secretion was similar on days 2 and 4, but decreased on day 6 (Figure 4b). Based on the in vivo experiments, we performed further validation in vitro. IHC staining of BMP2 in the skin of the defect area revealed significantly more positive cells (red arrow) in the FFA group after both 6 and 8 weeks of treatment (Figure 4c). Furthermore, there was a significantly larger area of positive staining in the mouse cranial bone in the presence of FFA treatment (BMP2) (Figure 4d).

## 4. Discussion

In the present study, we found that topical application of butyl flufenamate accelerated the healing of cranial defects in mice by promoting BMP2 secretion in mSMSCs. These findings suggest that butyl flufenamate ointment may have a therapeutic value for treating superficial fractures or bone defects while avoiding the toxicity and side effects of systemic medication, representing a safe and convenient adjuvant therapy to promote the healing of superficial bone defects and fractures.

We verified in previous studies that low concentrations of FFA can regulate osteogenic and adipogenic differentiation of human mesenchymal stem cells both in vivo and in vitro [14,15]. Systemic administration of FFA also suppressed bone loss in ovariectomized and aged mice [14]. However, oral systemic use of FFA is no longer recommended due to kidney and digestive tract damage [10,11,12,13], which limits the practical application of our previous research. Thus, we turned our attention to the external preparation of FFA using butyl flufenamate ointment. Butyl flufenamate ointment is a mature commercial drug approved by the FDA that is safe, convenient, and economical. The active component of butyl flufenamate ointment is FFA, up to 95% of which accumulates in the skin [16,17] and does not reach the subcutaneous area. We chose a mouse cranial-defect model for preliminary evaluation of butyl flufenamate ointment as an adjuvant to promote the healing of superficial bone defects or fractures.

This research is an extension and continuation of our previous studies in terms of its practical value. BMP2 is an endogenously secreted protein that belongs to the transforming growth factor (TGB)-β protein superfamily [21]. BMP2 has the ability to induce new bone formation in ectopic sites and has been proven to be a safe and effective bone-inducing growth factor [22,23,24,25]. We found that butyl flufenamate ointment and FFA promoted BMP2 secretion in mSMSCs both in vivo and in vitro. Butyl flufenamate ointment and FFA are generally believed to play an anti-inflammatory role [26,27,28]; we are the first to show that they can induce mSMSCs to secrete the key osteogenic factor BMP2 [29,30,31]. Although the mechanism of action by which the topical application of butyl flufenamate ointment accelerates healing of cranial defects in mice has not been completely elucidated, it is mediated by promoting BMP2 secretion in mSMSCs. We believe that the ointment stimulates BMP2 secreted by mSMSCs, which acts on the cranial bone below the skin and repairs the superficial cranial defects [23].

Our study still has several limitations. We used a mouse cranial defect model to assess the effects of external use of butyl flufenamate ointment; different bone defect/fracture models should be used to verify these effects. While we showed that butyl flufenamate ointment promoted BMP2 secretion in mSMSCs, further studies are required to comprehensively determine the mechanism of its effects.

Overall, our results demonstrated that the external use of butyl flufenamate ointment promoted the healing of cranial defects in mice, suggesting that it might have therapeutic value as an adjuvant for external use to promote healing of superficial bone defects and fractures.

## 5. Conclusions

The present study showed that topical application of butyl flufenamate ointment accelerates the healing of cranial defects in mice by promoting BMP2 secretion in mSMSCs. These findings suggest that butyl flufenamate ointment may have therapeutic value for treating superficial fractures or bone defects while avoiding the side effects of systemic medication, representing a safe and convenient adjuvant therapy.

## Data Availability

The authors confirm that all data underlying the findings are fully available.
